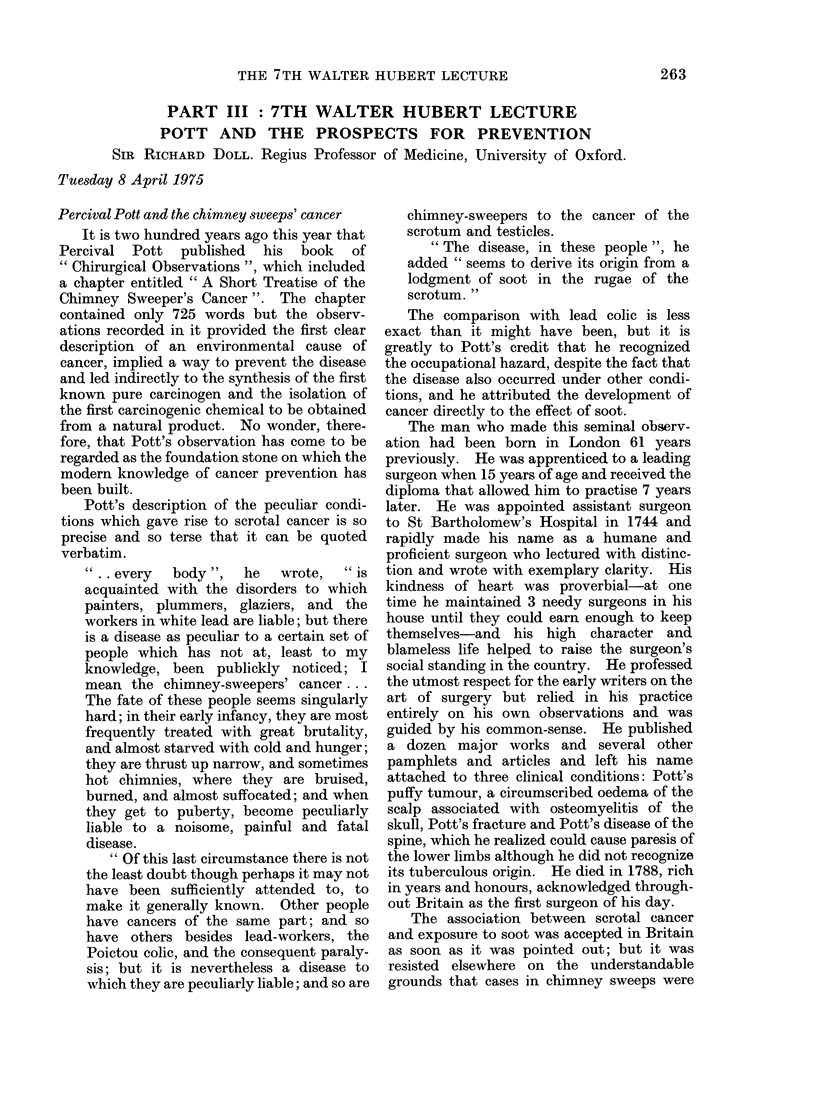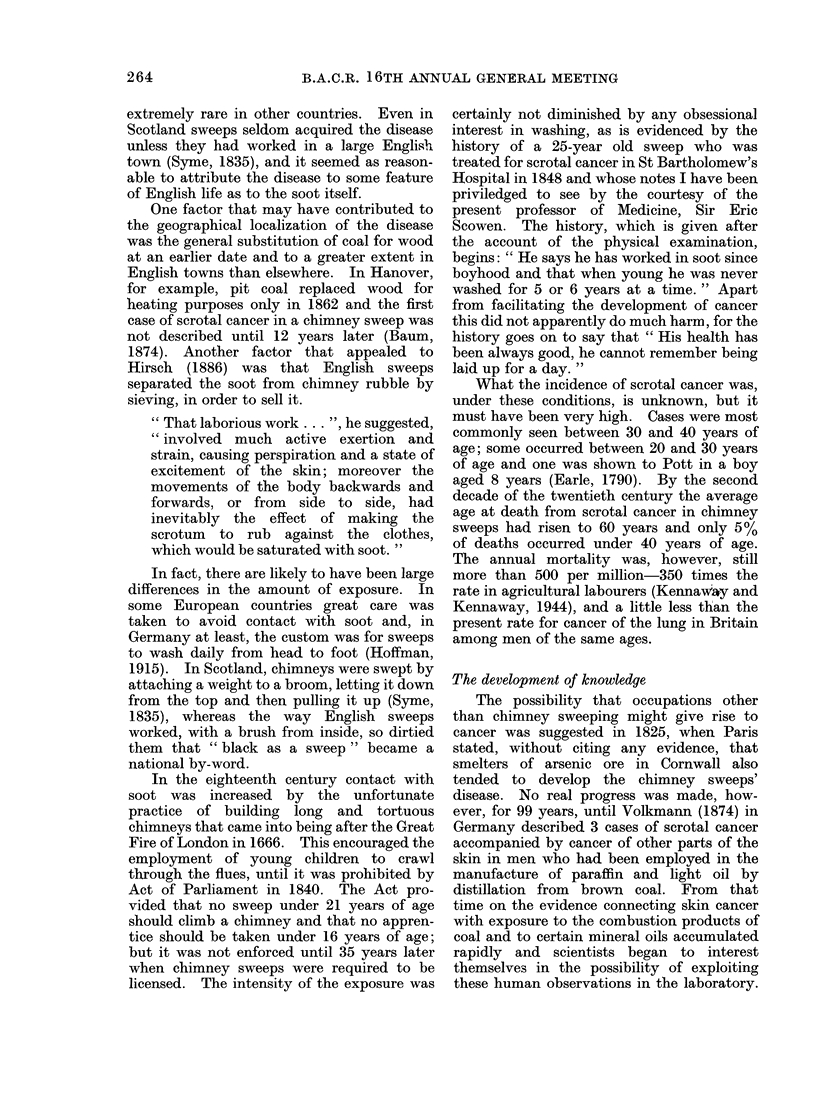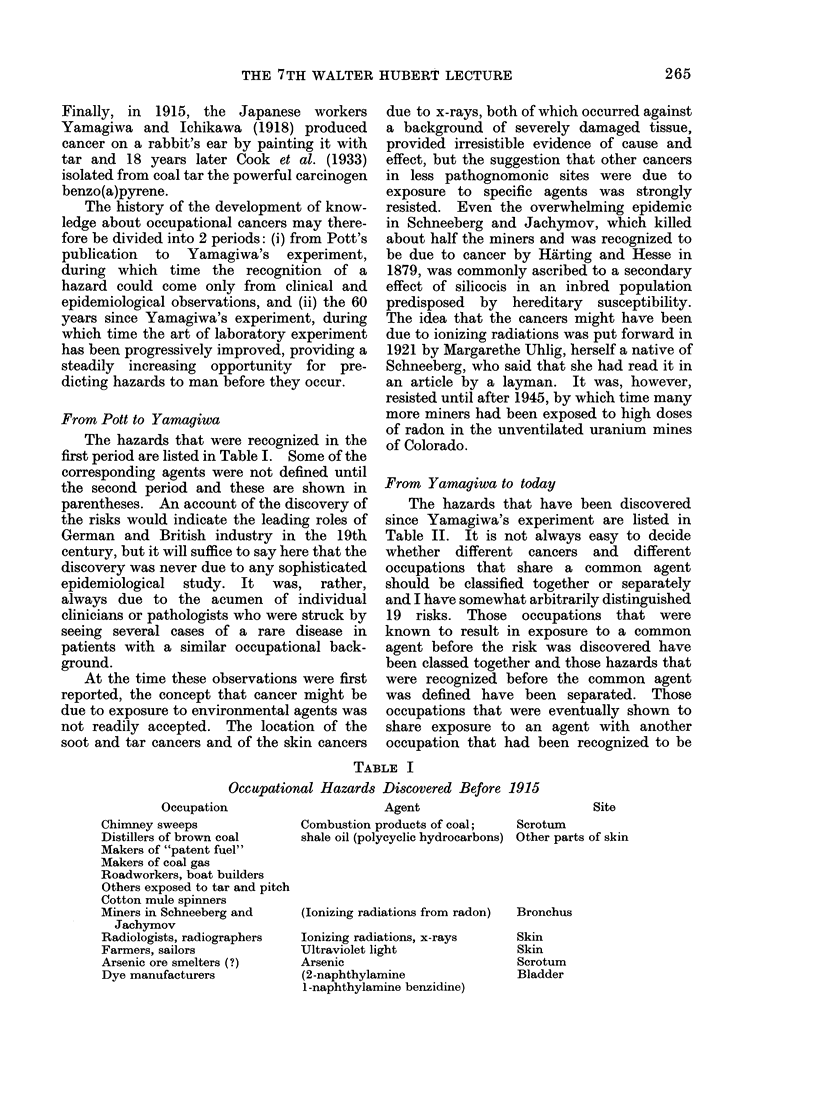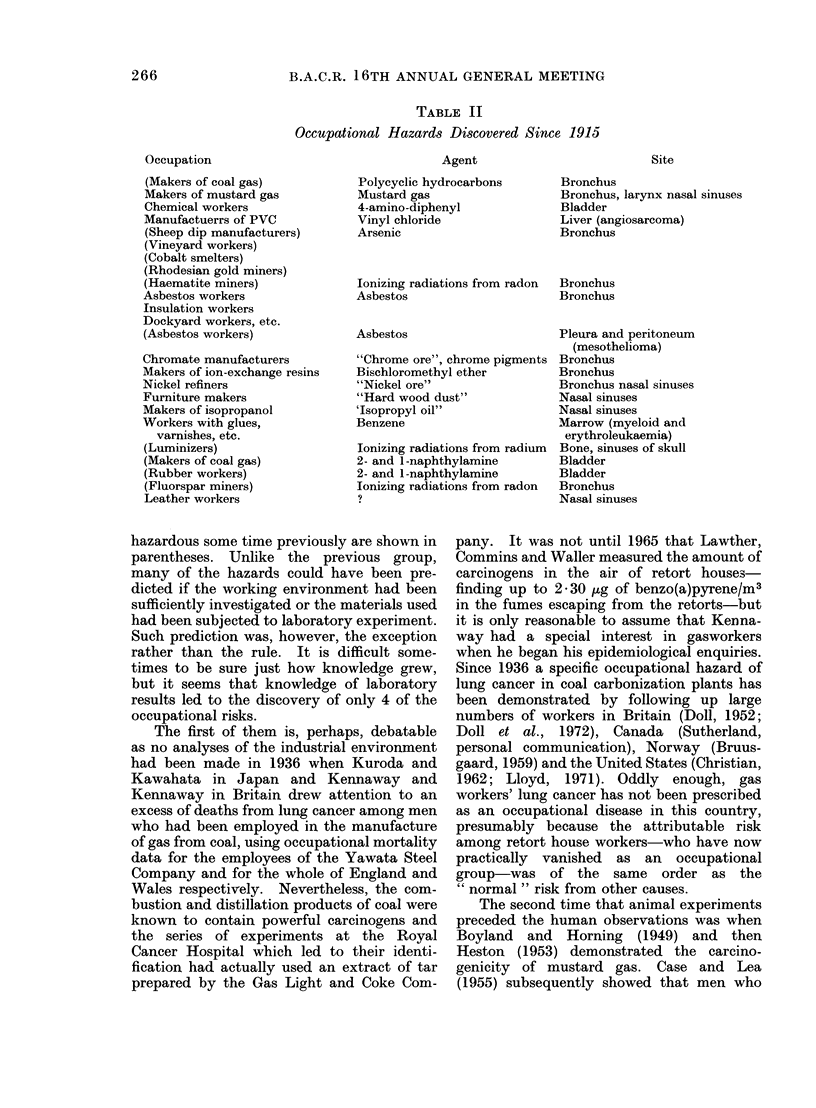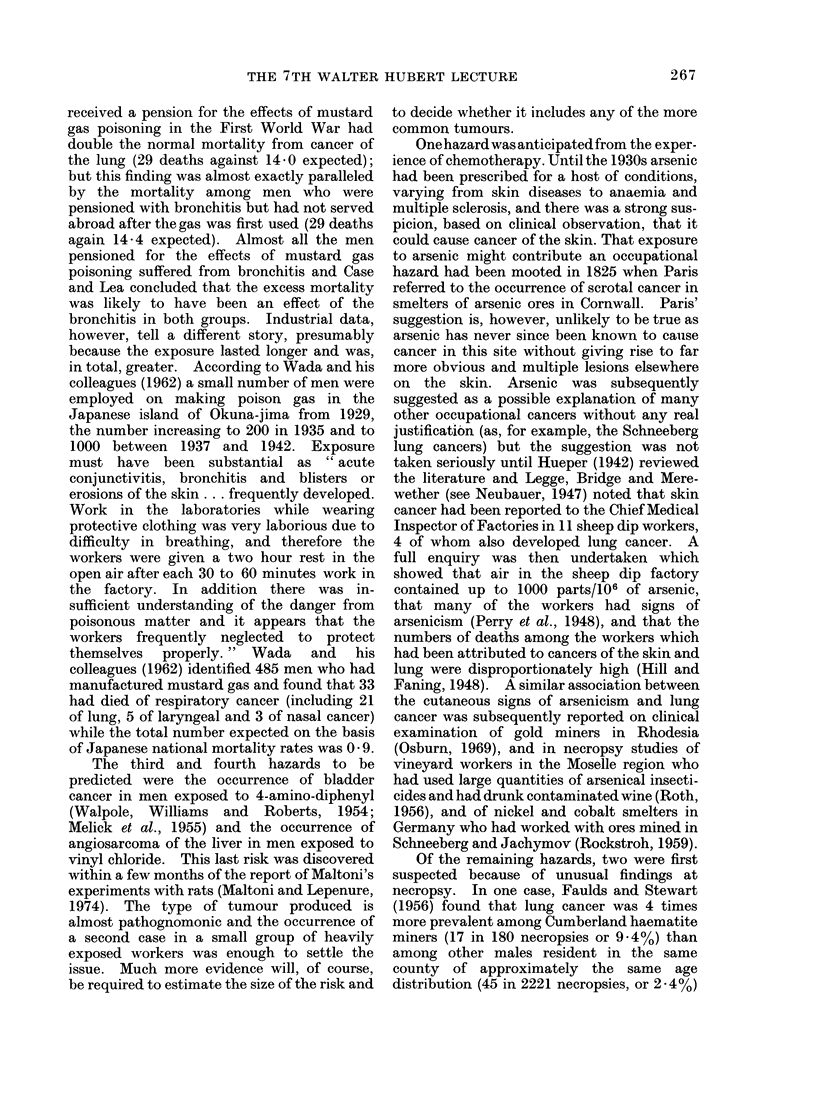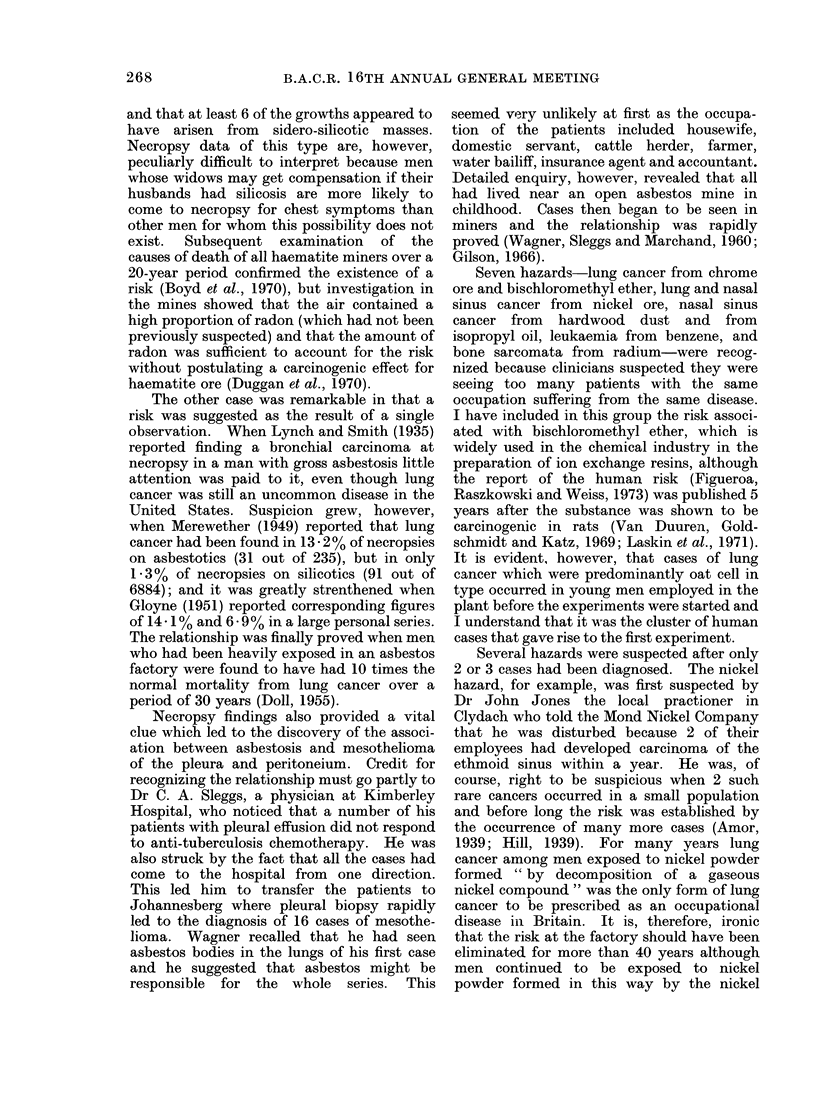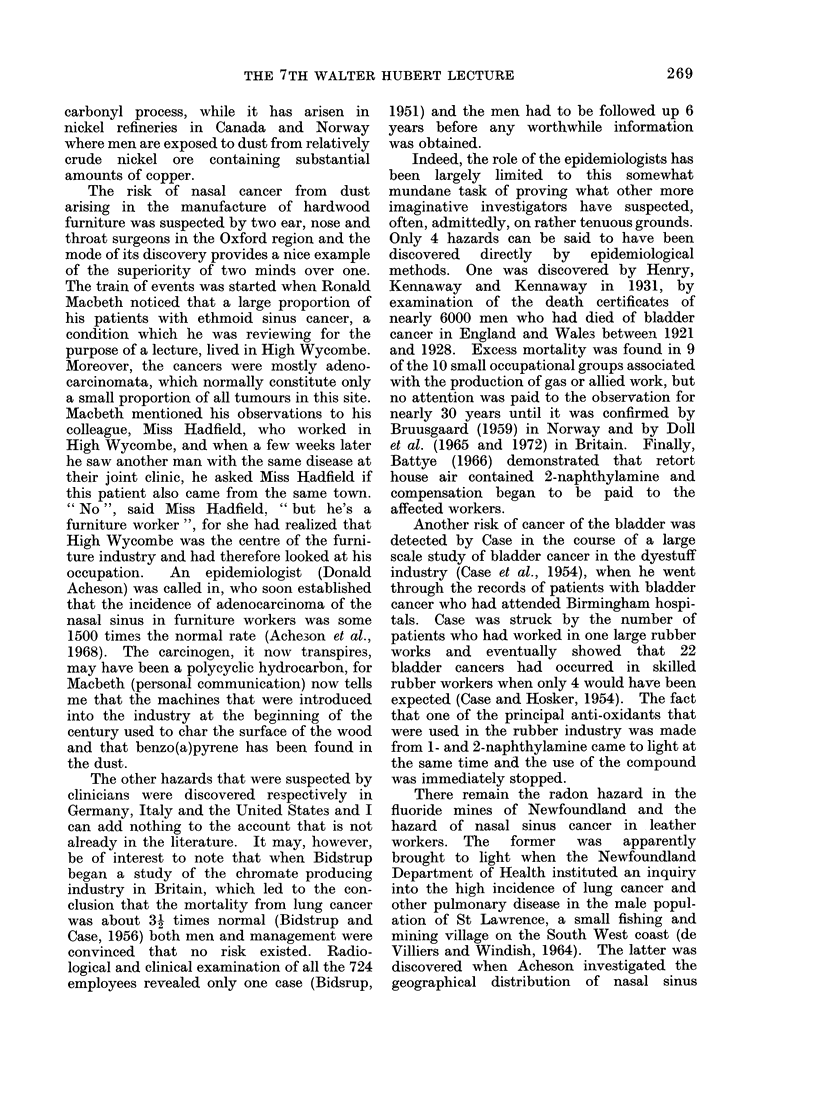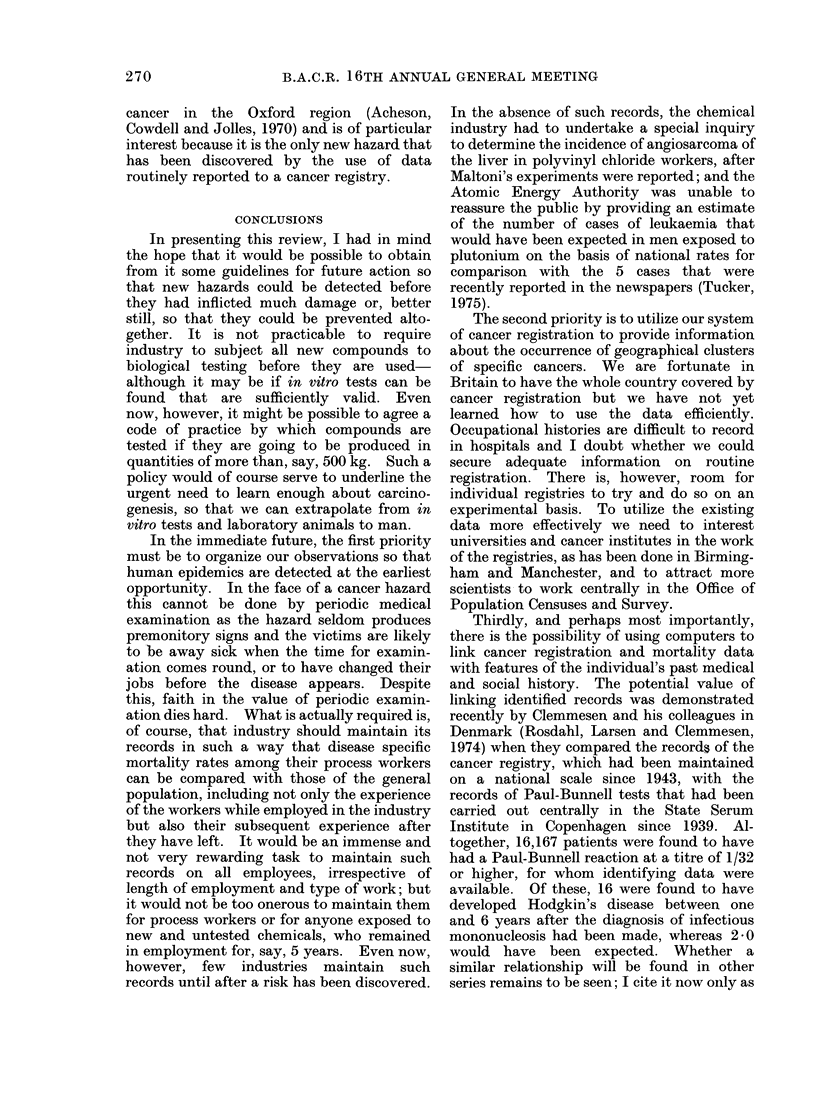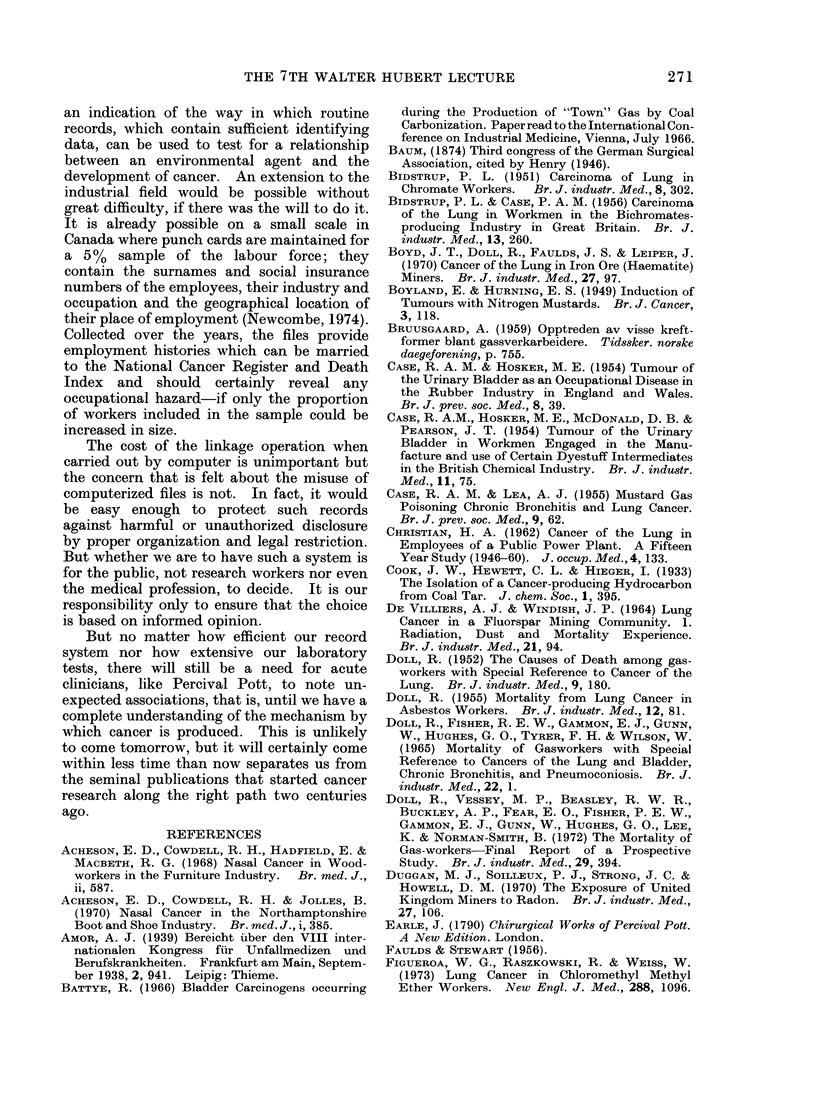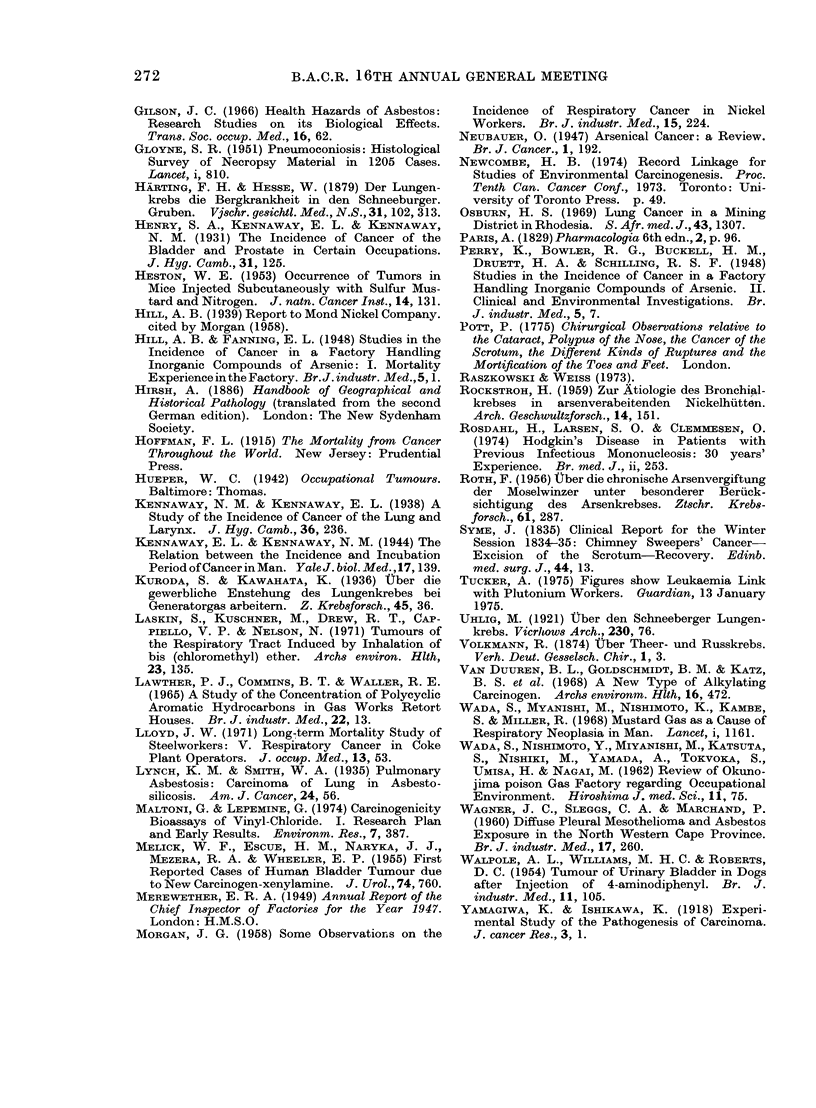# Part III: 7th Walter Hubert lecture. Pott and the prospects for prevention.

**DOI:** 10.1038/bjc.1975.212

**Published:** 1975-08

**Authors:** R. Doll


### PART III : 7TH WALTER HUBERT LECTURE
### POTT AND THE PROSPECTS FOR PREVENTION

SIR RICHARD DOLL. Regius Professor of Medicine, University of Oxford.

*Tuesday 8 April 1975*

*Percival Pott and the chimney sweeps' cancer*

It is two hundred years ago this year that Percival Pott published his book of " Chirurgical Observations ", which included a chapter entitled " A Short Treatise of the Chimney Sweeper's Cancer ". The chapter contained only 725 words but the observations recorded in it provided the first clear description of an environmental cause of cancer, implied a way to prevent the disease and led indirectly to the synthesis of the first known pure carcinogen and the isolation of the first carcinogenic chemical to be obtained from a natural product. No wonder, therefore, that Pott's observation has come to be regarded as the foundation stone on which the modern knowledge of cancer prevention has been built.

Pott's description of the peculiar conditions which gave rise to scrotal cancer is so precise and so terse that it can be quoted verbatim.

> " . . every body ", he wrote, " is acquainted with the disorders to which painters, plummers, glaziers, and the workers in white lead are liable; but there is a disease as peculiar to a certain set of people which has not at, least to my knowledge, been publickly noticed; I mean the chimney-sweepers' cancer . . . The fate of these people seems singularly hard; in their early infancy, they are most frequently treated with great brutality, and almost starved with cold and hunger; they are thrust up narrow, and sometimes hot chimnies, where they are bruised, burned, and almost suffocated; and when they get to puberty, become peculiarly liable to a noisome, painful and fatal disease.
>
> " Of this last circumstance there is not the least doubt though perhaps it may not have been sufficiently attended to, to make it generally known. Other people have cancers of the same part; and so have others besides lead-workers, the Poictou colic, and the consequent paralysis; but it is nevertheless a disease to which they are peculiarly liable; and so are chimney-sweepers to the cancer of the scrotum and testicles.
>
> " The disease, in these people ", he added " seems to derive its origin from a lodgment of soot in the rugae of the scrotum. "

The comparison with lead colic is less exact than it might have been, but it is greatly to Pott's credit that he recognized the occupational hazard, despite the fact that the disease also occurred under other conditions, and he attributed the development of cancer directly to the effect of soot.

The man who made this seminal observation had been born in London 61 years previously. He was apprenticed to a leading surgeon when 15 years of age and received the diploma that allowed him to practise 7 years later. He was appointed assistant surgeon to St Bartholomew's Hospital in 1744 and rapidly made his name as a humane and proficient surgeon who lectured with distinction and wrote with exemplary clarity. His kindness of heart was proverbial—at one time he maintained 3 needy surgeons in his house until they could earn enough to keep themselves—and his high character and blameless life helped to raise the surgeon's social standing in the country. He professed the utmost respect for the early writers on the art of surgery but relied in his practice entirely on his own observations and was guided by his common-sense. He published a dozen major works and several other pamphlets and articles and left his name attached to three clinical conditions: Pott's puffy tumour, a circumscribed oedema of the scalp associated with osteomyelitis of the skull, Pott's fracture and Pott's disease of the spine, which he realized could cause paresis of the lower limbs although he did not recognize its tuberculous origin. He died in 1788, rich in years and honours, acknowledged throughout Britain as the first surgeon of his day.

The association between scrotal cancer and exposure to soot was accepted in Britain as soon as it was pointed out; but it was resisted elsewhere on the understandable grounds that cases in chimney sweeps were extremely rare in other countries. Even in Scotland sweeps seldom acquired the disease unless they had worked in a large English town (Syme, 1835), and it seemed as reasonable to attribute the disease to some feature of English life as to the soot itself.

One factor that may have contributed to the geographical localization of the disease was the general substitution of coal for wood at an earlier date and to a greater extent in English towns than elsewhere. In Hanover, for example, pit coal replaced wood for heating purposes only in 1862 and the first case of scrotal cancer in a chimney sweep was not described until 12 years later (Baum, 1874). Another factor that appealed to Hirsch (1886) was that English sweeps separated the soot from chimney rubble by sieving, in order to sell it.

> " That laborious work . . . ", he suggested, " involved much active exertion and strain, causing perspiration and a state of excitement of the skin; moreover the movements of the body backwards and forwards, or from side to side, had inevitably the effect of making the scrotum to rub against the clothes, which would be saturated with soot. "

In fact, there are likely to have been large differences in the amount of exposure. In some European countries great care was taken to avoid contact with soot and, in Germany at least, the custom was for sweeps to wash daily from head to foot (Hoffman, 1915). In Scotland, chimneys were swept by attaching a weight to a broom, letting it down from the top and then pulling it up (Syme, 1835), whereas the way English sweeps worked, with a brush from inside, so dirtied them that " black as a sweep " became a national by-word.

In the eighteenth century contact with soot was increased by the unfortunate practice of building long and tortuous chimneys that came into being after the Great Fire of London in 1666. This encouraged the employment of young children to crawl through the flues, until it was prohibited by Act of Parliament in 1840. The Act provided that no sweep under 21 years of age should climb a chimney and that no apprentice should be taken under 16 years of age; but it was not enforced until 35 years later when chimney sweeps were required to be licensed. The intensity of the exposure was certainly not diminished by any obsessional interest in washing, as is evidenced by the history of a 25-year old sweep who was treated for scrotal cancer in St Bartholomew's Hospital in 1848 and whose notes I have been priviledged to see by the courtesy of the present professor of Medicine, Sir Eric Scowen. The history, which is given after the account of the physical examination, begins: " He says he has worked in soot since boyhood and that when young he was never washed for 5 or 6 years at a time. " Apart from facilitating the development of cancer this did not apparently do much harm, for the history goes on to say that " His health has been always good, he cannot remember being laid up for a day. "

What the incidence of scrotal cancer was, under these conditions, is unknown, but it must have been very high. Cases were most commonly seen between 30 and 40 years of age; some occurred between 20 and 30 years of age and one was shown to Pott in a boy aged 8 years (Earle, 1790). By the second decade of the twentieth century the average age at death from scrotal cancer in chimney sweeps had risen to 60 years and only 5% of deaths occurred under 40 years of age. The annual mortality was, however, still more than 500 per million—350 times the rate in agricultural labourers (Kennaway and Kennaway, 1944), and a little less than the present rate for cancer of the lung in Britain among men of the same ages.

### *The development of knowledge*

The possibility that occupations other than chimney sweeping might give rise to cancer was suggested in 1825, when Paris stated, without citing any evidence, that smelters of arsenic ore in Cornwall also tended to develop the chimney sweeps' disease. No real progress was made, however, for 99 years, until Volkmann (1874) in Germany described 3 cases of scrotal cancer accompanied by cancer of other parts of the skin in men who had been employed in the manufacture of paraffin and light oil by distillation from brown coal. From that time on the evidence connecting skin cancer with exposure to the combustion products of coal and to certain mineral oils accumulated rapidly and scientists began to interest themselves in the possibility of exploiting these human observations in the laboratory.

Finally, in 1915, the Japanese workers Yamagiwa and Ichikawa (1918) produced cancer on a rabbit's ear by painting it with tar and 18 years later Cook *et al.* (1933) isolated from coal tar the powerful carcinogen benzo(a)pyrene.

The history of the development of knowledge about occupational cancers may therefore be divided into 2 periods: (i) from Pott's publication to Yamagiwa's experiment, during which time the recognition of a hazard could come only from clinical and epidemiological observations, and (ii) the 60 years since Yamagiwa's experiment, during which time the art of laboratory experiment has been progressively improved, providing a steadily increasing opportunity for predicting hazards to man before they occur.

*From Pott to Yamagiwa*

The hazards that were recognized in the first period are listed in Table I. Some of the corresponding agents were not defined until the second period and these are shown in parentheses. An account of the discovery of the risks would indicate the leading roles of German and British industry in the 19th century, but it will suffice to say here that the discovery was never due to any sophisticated epidemiological study. It was, rather, always due to the acumen of individual clinicians or pathologists who were struck by seeing several cases of a rare disease in patients with a similar occupational background.

At the time these observations were first reported, the concept that cancer might be due to exposure to environmental agents was not readily accepted. The location of the soot and tar cancers and of the skin cancers due to x-rays, both of which occurred against a background of severely damaged tissue, provided irresistible evidence of cause and effect, but the suggestion that other cancers in less pathognomonic sites were due to exposure to specific agents was strongly resisted. Even the overwhelming epidemic in Schneeberg and Jachymov, which killed about half the miners and was recognized to be due to cancer by Härting and Hesse in 1879, was commonly ascribed to a secondary effect of silicocis in an inbred population predisposed by hereditary susceptibility. The idea that the cancers might have been due to ionizing radiations was put forward in 1921 by Margarethe Uhlig, herself a native of Schneeberg, who said that she had read it in an article by a layman. It was, however, resisted until after 1945, by which time many more miners had been exposed to high doses of radon in the unventilated uranium mines of Colorado.

*From Yamagiwa to today*

The hazards that have been discovered since Yamagiwa's experiment are listed in Table II. It is not always easy to decide whether different cancers and different occupations that share a common agent should be classified together or separately and I have somewhat arbitrarily distinguished 19 risks. Those occupations that were known to result in exposure to a common agent before the risk was discovered have been classed together and those hazards that were recognized before the common agent was defined have been separated. Those occupations that were eventually shown to share exposure to an agent with another occupation that had been recognized to be

TABLE I

*Occupational Hazards Discovered Before 1915*

| Occupation | Agent | Site |
|---|---|---|
| Chimney sweeps<br>Distillers of brown coal<br>Makers of "patent fuel"<br>Makers of coal gas<br>Roadworkers, boat builders<br>Others exposed to tar and pitch<br>Cotton mule spinners | Combustion products of coal;<br>shale oil (polycyclic hydrocarbons) | Scrotum<br>Other parts of skin |
| Miners in Schneeberg and Jachymov | (Ionizing radiations from radon) | Bronchus |
| Radiologists, radiographers | Ionizing radiations, x-rays | Skin |
| Farmers, sailors | Ultraviolet light | Skin |
| Arsenic ore smelters (?) | Arsenic | Scrotum |
| Dye manufacturers | (2-naphthylamine<br>1-naphthylamine benzidine) | Bladder |

TABLE II

*Occupational Hazards Discovered Since 1915*

| Occupation | Agent | Site |
|---|---|---|
| (Makers of coal gas) | Polycyclic hydrocarbons | Bronchus |
| Makers of mustard gas | Mustard gas | Bronchus, larynx nasal sinuses |
| Chemical workers | 4-amino-diphenyl | Bladder |
| Manufactuerrs of PVC | Vinyl chloride | Liver (angiosarcoma) |
| (Sheep dip manufacturers) (Vineyard workers) (Cobalt smelters) (Rhodesian gold miners) | Arsenic | Bronchus |
| (Haematite miners) | Ionizing radiations from radon | Bronchus |
| Asbestos workers Insulation workers Dockyard workers, etc. | Asbestos | Bronchus |
| (Asbestos workers) | Asbestos | Pleura and peritoneum (mesothelioma) |
| Chromate manufacturers | "Chrome ore", chrome pigments | Bronchus |
| Makers of ion-exchange resins | Bischloromethyl ether | Bronchus |
| Nickel refiners | "Nickel ore" | Bronchus nasal sinuses |
| Furniture makers | "Hard wood dust" | Nasal sinuses |
| Makers of isopropanol | 'Isopropyl oil" | Nasal sinuses |
| Workers with glues, varnishes, etc. | Benzene | Marrow (myeloid and erythroleukaemia) |
| (Luminizers) | Ionizing radiations from radium | Bone, sinuses of skull |
| (Makers of coal gas) | 2- and 1-naphthylamine | Bladder |
| (Rubber workers) | 2- and 1-naphthylamine | Bladder |
| (Fluorspar miners) | Ionizing radiations from radon | Bronchus |
| Leather workers | ? | Nasal sinuses |

hazardous some time previously are shown in parentheses. Unlike the previous group, many of the hazards could have been predicted if the working environment had been sufficiently investigated or the materials used had been subjected to laboratory experiment. Such prediction was, however, the exception rather than the rule. It is difficult sometimes to be sure just how knowledge grew, but it seems that knowledge of laboratory results led to the discovery of only 4 of the occupational risks.

The first of them is, perhaps, debatable as no analyses of the industrial environment had been made in 1936 when Kuroda and Kawahata in Japan and Kennaway and Kennaway in Britain drew attention to an excess of deaths from lung cancer among men who had been employed in the manufacture of gas from coal, using occupational mortality data for the employees of the Yawata Steel Company and for the whole of England and Wales respectively. Nevertheless, the combustion and distillation products of coal were known to contain powerful carcinogens and the series of experiments at the Royal Cancer Hospital which led to their identification had actually used an extract of tar prepared by the Gas Light and Coke Company. It was not until 1965 that Lawther, Commins and Waller measured the amount of carcinogens in the air of retort houses—finding up to 2·30 $\mu$g of benzo(a)pyrene/m$^3$ in the fumes escaping from the retorts—but it is only reasonable to assume that Kennaway had a special interest in gasworkers when he began his epidemiological enquiries. Since 1936 a specific occupational hazard of lung cancer in coal carbonization plants has been demonstrated by following up large numbers of workers in Britain (Doll, 1952; Doll *et al.*, 1972), Canada (Sutherland, personal communication), Norway (Bruusgaard, 1959) and the United States (Christian, 1962; Lloyd, 1971). Oddly enough, gas workers' lung cancer has not been prescribed as an occupational disease in this country, presumably because the attributable risk among retort house workers—who have now practically vanished as an occupational group—was of the same order as the " normal " risk from other causes.

The second time that animal experiments preceded the human observations was when Boyland and Horning (1949) and then Heston (1953) demonstrated the carcinogenicity of mustard gas. Case and Lea (1955) subsequently showed that men who received a pension for the effects of mustard gas poisoning in the First World War had double the normal mortality from cancer of the lung (29 deaths against 14·0 expected); but this finding was almost exactly paralleled by the mortality among men who were pensioned with bronchitis but had not served abroad after the gas was first used (29 deaths again 14·4 expected). Almost all the men pensioned for the effects of mustard gas poisoning suffered from bronchitis and Case and Lea concluded that the excess mortality was likely to have been an effect of the bronchitis in both groups. Industrial data, however, tell a different story, presumably because the exposure lasted longer and was, in total, greater. According to Wada and his colleagues (1962) a small number of men were employed on making poison gas in the Japanese island of Okuna-jima from 1929, the number increasing to 200 in 1935 and to 1000 between 1937 and 1942. Exposure must have been substantial as "acute conjunctivitis, bronchitis and blisters or erosions of the skin . . . frequently developed. Work in the laboratories while wearing protective clothing was very laborious due to difficulty in breathing, and therefore the workers were given a two hour rest in the open air after each 30 to 60 minutes work in the factory. In addition there was insufficient understanding of the danger from poisonous matter and it appears that the workers frequently neglected to protect themselves properly." Wada and his colleagues (1962) identified 485 men who had manufactured mustard gas and found that 33 had died of respiratory cancer (including 21 of lung, 5 of laryngeal and 3 of nasal cancer) while the total number expected on the basis of Japanese national mortality rates was 0·9.

The third and fourth hazards to be predicted were the occurrence of bladder cancer in men exposed to 4-amino-diphenyl (Walpole, Williams and Roberts, 1954; Melick *et al.*, 1955) and the occurrence of angiosarcoma of the liver in men exposed to vinyl chloride. This last risk was discovered within a few months of the report of Maltoni's experiments with rats (Maltoni and Lepenure, 1974). The type of tumour produced is almost pathognomonic and the occurrence of a second case in a small group of heavily exposed workers was enough to settle the issue. Much more evidence will, of course, be required to estimate the size of the risk and to decide whether it includes any of the more common tumours.

One hazard was anticipated from the experience of chemotherapy. Until the 1930s arsenic had been prescribed for a host of conditions, varying from skin diseases to anaemia and multiple sclerosis, and there was a strong suspicion, based on clinical observation, that it could cause cancer of the skin. That exposure to arsenic might contribute an occupational hazard had been mooted in 1825 when Paris referred to the occurrence of scrotal cancer in smelters of arsenic ores in Cornwall. Paris' suggestion is, however, unlikely to be true as arsenic has never since been known to cause cancer in this site without giving rise to far more obvious and multiple lesions elsewhere on the skin. Arsenic was subsequently suggested as a possible explanation of many other occupational cancers without any real justification (as, for example, the Schneeberg lung cancers) but the suggestion was not taken seriously until Hueper (1942) reviewed the literature and Legge, Bridge and Merewether (see Neubauer, 1947) noted that skin cancer had been reported to the Chief Medical Inspector of Factories in 11 sheep dip workers, 4 of whom also developed lung cancer. A full enquiry was then undertaken which showed that air in the sheep dip factory contained up to 1000 parts/$10^6$ of arsenic, that many of the workers had signs of arsenicism (Perry *et al.*, 1948), and that the numbers of deaths among the workers which had been attributed to cancers of the skin and lung were disproportionately high (Hill and Faning, 1948). A similar association between the cutaneous signs of arsenicism and lung cancer was subsequently reported on clinical examination of gold miners in Rhodesia (Osburn, 1969), and in necropsy studies of vineyard workers in the Moselle region who had used large quantities of arsenical insecticides and had drunk contaminated wine (Roth, 1956), and of nickel and cobalt smelters in Germany who had worked with ores mined in Schneeberg and Jachymov (Rockstroh, 1959).

Of the remaining hazards, two were first suspected because of unusual findings at necropsy. In one case, Faulds and Stewart (1956) found that lung cancer was 4 times more prevalent among Cumberland haematite miners (17 in 180 necropsies or 9·4%) than among other males resident in the same county of approximately the same age distribution (45 in 2221 necropsies, or 2·4%)

and that at least 6 of the growths appeared to have arisen from sidero-silicotic masses. Necropsy data of this type are, however, peculiarly difficult to interpret because men whose widows may get compensation if their husbands had silicosis are more likely to come to necropsy for chest symptoms than other men for whom this possibility does not exist. Subsequent examination of the causes of death of all haematite miners over a 20-year period confirmed the existence of a risk (Boyd *et al.*, 1970), but investigation in the mines showed that the air contained a high proportion of radon (which had not been previously suspected) and that the amount of radon was sufficient to account for the risk without postulating a carcinogenic effect for haematite ore (Duggan *et al.*, 1970).

The other case was remarkable in that a risk was suggested as the result of a single observation. When Lynch and Smith (1935) reported finding a bronchial carcinoma at necropsy in a man with gross asbestosis little attention was paid to it, even though lung cancer was still an uncommon disease in the United States. Suspicion grew, however, when Merewether (1949) reported that lung cancer had been found in 13·2% of necropsies on asbestotics (31 out of 235), but in only 1·3% of necropsies on silicotics (91 out of 6884); and it was greatly strenthened when Gloyne (1951) reported corresponding figures of 14·1% and 6·9% in a large personal series. The relationship was finally proved when men who had been heavily exposed in an asbestos factory were found to have had 10 times the normal mortality from lung cancer over a period of 30 years (Doll, 1955).

Necropsy findings also provided a vital clue which led to the discovery of the association between asbestosis and mesothelioma of the pleura and peritoneium. Credit for recognizing the relationship must go partly to Dr C. A. Sleggs, a physician at Kimberley Hospital, who noticed that a number of his patients with pleural effusion did not respond to anti-tuberculosis chemotherapy. He was also struck by the fact that all the cases had come to the hospital from one direction. This led him to transfer the patients to Johannesberg where pleural biopsy rapidly led to the diagnosis of 16 cases of mesothelioma. Wagner recalled that he had seen asbestos bodies in the lungs of his first case and he suggested that asbestos might be responsible for the whole series. This seemed very unlikely at first as the occupation of the patients included housewife, domestic servant, cattle herder, farmer, water bailiff, insurance agent and accountant. Detailed enquiry, however, revealed that all had lived near an open asbestos mine in childhood. Cases then began to be seen in miners and the relationship was rapidly proved (Wagner, Sleggs and Marchand, 1960; Gilson, 1966).

Seven hazards—lung cancer from chrome ore and bischloromethyl ether, lung and nasal sinus cancer from nickel ore, nasal sinus cancer from hardwood dust and from isopropyl oil, leukaemia from benzene, and bone sarcomata from radium—were recognized because clinicians suspected they were seeing too many patients with the same occupation suffering from the same disease. I have included in this group the risk associated with bischloromethyl ether, which is widely used in the chemical industry in the preparation of ion exchange resins, although the report of the human risk (Figueroa, Raszkowski and Weiss, 1973) was published 5 years after the substance was shown to be carcinogenic in rats (Van Duuren, Goldschmidt and Katz, 1969; Laskin *et al.*, 1971). It is evident, however, that cases of lung cancer which were predominantly oat cell in type occurred in young men employed in the plant before the experiments were started and I understand that it was the cluster of human cases that gave rise to the first experiment.

Several hazards were suspected after only 2 or 3 cases had been diagnosed. The nickel hazard, for example, was first suspected by Dr John Jones the local practioner in Clydach who told the Mond Nickel Company that he was disturbed because 2 of their employees had developed carcinoma of the ethmoid sinus within a year. He was, of course, right to be suspicious when 2 such rare cancers occurred in a small population and before long the risk was established by the occurrence of many more cases (Amor, 1939; Hill, 1939). For many years lung cancer among men exposed to nickel powder formed " by decomposition of a gaseous nickel compound " was the only form of lung cancer to be prescribed as an occupational disease in Britain. It is, therefore, ironic that the risk at the factory should have been eliminated for more than 40 years although men continued to be exposed to nickel powder formed in this way by the nickel carbonyl process, while it has arisen in nickel refineries in Canada and Norway where men are exposed to dust from relatively crude nickel ore containing substantial amounts of copper.

The risk of nasal cancer from dust arising in the manufacture of hardwood furniture was suspected by two ear, nose and throat surgeons in the Oxford region and the mode of its discovery provides a nice example of the superiority of two minds over one. The train of events was started when Ronald Macbeth noticed that a large proportion of his patients with ethmoid sinus cancer, a condition which he was reviewing for the purpose of a lecture, lived in High Wycombe. Moreover, the cancers were mostly adeno-carcinomata, which normally constitute only a small proportion of all tumours in this site. Macbeth mentioned his observations to his colleague, Miss Hadfield, who worked in High Wycombe, and when a few weeks later he saw another man with the same disease at their joint clinic, he asked Miss Hadfield if this patient also came from the same town. " No ", said Miss Hadfield, " but he's a furniture worker ", for she had realized that High Wycombe was the centre of the furniture industry and had therefore looked at his occupation. An epidemiologist (Donald Acheson) was called in, who soon established that the incidence of adenocarcinoma of the nasal sinus in furniture workers was some 1500 times the normal rate (Acheson *et al.*, 1968). The carcinogen, it now transpires, may have been a polycyclic hydrocarbon, for Macbeth (personal communication) now tells me that the machines that were introduced into the industry at the beginning of the century used to char the surface of the wood and that benzo(a)pyrene has been found in the dust.

The other hazards that were suspected by clinicians were discovered respectively in Germany, Italy and the United States and I can add nothing to the account that is not already in the literature. It may, however, be of interest to note that when Bidstrup began a study of the chromate producing industry in Britain, which led to the conclusion that the mortality from lung cancer was about $3\frac{1}{2}$ times normal (Bidstrup and Case, 1956) both men and management were convinced that no risk existed. Radiological and clinical examination of all the 724 employees revealed only one case (Bidsrup, 1951) and the men had to be followed up 6 years before any worthwhile information was obtained.

Indeed, the role of the epidemiologists has been largely limited to this somewhat mundane task of proving what other more imaginative investigators have suspected, often, admittedly, on rather tenuous grounds. Only 4 hazards can be said to have been discovered directly by epidemiological methods. One was discovered by Henry, Kennaway and Kennaway in 1931, by examination of the death certificates of nearly 6000 men who had died of bladder cancer in England and Wales between 1921 and 1928. Excess mortality was found in 9 of the 10 small occupational groups associated with the production of gas or allied work, but no attention was paid to the observation for nearly 30 years until it was confirmed by Bruusgaard (1959) in Norway and by Doll *et al.* (1965 and 1972) in Britain. Finally, Battye (1966) demonstrated that retort house air contained 2-naphthylamine and compensation began to be paid to the affected workers.

Another risk of cancer of the bladder was detected by Case in the course of a large scale study of bladder cancer in the dyestuff industry (Case *et al.*, 1954), when he went through the records of patients with bladder cancer who had attended Birmingham hospitals. Case was struck by the number of patients who had worked in one large rubber works and eventually showed that 22 bladder cancers had occurred in skilled rubber workers when only 4 would have been expected (Case and Hosker, 1954). The fact that one of the principal anti-oxidants that were used in the rubber industry was made from 1- and 2-naphthylamine came to light at the same time and the use of the compound was immediately stopped.

There remain the radon hazard in the fluoride mines of Newfoundland and the hazard of nasal sinus cancer in leather workers. The former was apparently brought to light when the Newfoundland Department of Health instituted an inquiry into the high incidence of lung cancer and other pulmonary disease in the male population of St Lawrence, a small fishing and mining village on the South West coast (de Villiers and Windish, 1964). The latter was discovered when Acheson investigated the geographical distribution of nasal sinus cancer in the Oxford region (Acheson, Cowdell and Jolles, 1970) and is of particular interest because it is the only new hazard that has been discovered by the use of data routinely reported to a cancer registry.

### CONCLUSIONS

In presenting this review, I had in mind the hope that it would be possible to obtain from it some guidelines for future action so that new hazards could be detected before they had inflicted much damage or, better still, so that they could be prevented altogether. It is not practicable to require industry to subject all new compounds to biological testing before they are used—although it may be if *in vitro* tests can be found that are sufficiently valid. Even now, however, it might be possible to agree a code of practice by which compounds are tested if they are going to be produced in quantities of more than, say, 500 kg. Such a policy would of course serve to underline the urgent need to learn enough about carcinogenesis, so that we can extrapolate from *in vitro* tests and laboratory animals to man.

In the immediate future, the first priority must be to organize our observations so that human epidemics are detected at the earliest opportunity. In the face of a cancer hazard this cannot be done by periodic medical examination as the hazard seldom produces premonitory signs and the victims are likely to be away sick when the time for examination comes round, or to have changed their jobs before the disease appears. Despite this, faith in the value of periodic examination dies hard. What is actually required is, of course, that industry should maintain its records in such a way that disease specific mortality rates among their process workers can be compared with those of the general population, including not only the experience of the workers while employed in the industry but also their subsequent experience after they have left. It would be an immense and not very rewarding task to maintain such records on all employees, irrespective of length of employment and type of work; but it would not be too onerous to maintain them for process workers or for anyone exposed to new and untested chemicals, who remained in employment for, say, 5 years. Even now, however, few industries maintain such records until after a risk has been discovered. In the absence of such records, the chemical industry had to undertake a special inquiry to determine the incidence of angiosarcoma of the liver in polyvinyl chloride workers, after Maltoni's experiments were reported; and the Atomic Energy Authority was unable to reassure the public by providing an estimate of the number of cases of leukaemia that would have been expected in men exposed to plutonium on the basis of national rates for comparison with the 5 cases that were recently reported in the newspapers (Tucker, 1975).

The second priority is to utilize our system of cancer registration to provide information about the occurrence of geographical clusters of specific cancers. We are fortunate in Britain to have the whole country covered by cancer registration but we have not yet learned how to use the data efficiently. Occupational histories are difficult to record in hospitals and I doubt whether we could secure adequate information on routine registration. There is, however, room for individual registries to try and do so on an experimental basis. To utilize the existing data more effectively we need to interest universities and cancer institutes in the work of the registries, as has been done in Birmingham and Manchester, and to attract more scientists to work centrally in the Office of Population Censuses and Survey.

Thirdly, and perhaps most importantly, there is the possibility of using computers to link cancer registration and mortality data with features of the individual's past medical and social history. The potential value of linking identified records was demonstrated recently by Clemmesen and his colleagues in Denmark (Rosdahl, Larsen and Clemmesen, 1974) when they compared the records of the cancer registry, which had been maintained on a national scale since 1943, with the records of Paul-Bunnell tests that had been carried out centrally in the State Serum Institute in Copenhagen since 1939. Altogether, 16,167 patients were found to have had a Paul-Bunnell reaction at a titre of 1/32 or higher, for whom identifying data were available. Of these, 16 were found to have developed Hodgkin's disease between one and 6 years after the diagnosis of infectious mononucleosis had been made, whereas 2·0 would have been expected. Whether a similar relationship will be found in other series remains to be seen; I cite it now only as an indication of the way in which routine records, which contain sufficient identifying data, can be used to test for a relationship between an environmental agent and the development of cancer. An extension to the industrial field would be possible without great difficulty, if there was the will to do it. It is already possible on a small scale in Canada where punch cards are maintained for a 5% sample of the labour force; they contain the surnames and social insurance numbers of the employees, their industry and occupation and the geographical location of their place of employment (Newcombe, 1974). Collected over the years, the files provide employment histories which can be married to the National Cancer Register and Death Index and should certainly reveal any occupational hazard—if only the proportion of workers included in the sample could be increased in size.

The cost of the linkage operation when carried out by computer is unimportant but the concern that is felt about the misuse of computerized files is not. In fact, it would be easy enough to protect such records against harmful or unauthorized disclosure by proper organization and legal restriction. But whether we are to have such a system is for the public, not research workers nor even the medical profession, to decide. It is our responsibility only to ensure that the choice is based on informed opinion.

But no matter how efficient our record system nor how extensive our laboratory tests, there will still be a need for acute clinicians, like Percival Pott, to note unexpected associations, that is, until we have a complete understanding of the mechanism by which cancer is produced. This is unlikely to come tomorrow, but it will certainly come within less time than now separates us from the seminal publications that started cancer research along the right path two centuries ago.

### REFERENCES

Acheson, E. D., Cowdell, R. H., Hadfield, E. & Macbeth, R. G. (1968) Nasal Cancer in Wood-workers in the Furniture Industry. *Br. med. J.*, ii, 587.

Acheson, E. D., Cowdell, R. H. & Jolles, B. (1970) Nasal Cancer in the Northamptonshire Boot and Shoe Industry. *Br. med. J.*, i, 385.

Amor, A. J. (1939) Bereicht über den VIII internationalen Kongress für Unfallmedizen und Berufskrankheiten. Frankfurt am Main, September 1938, **2**, 941. Leipig: Thieme.

Battye, R. (1966) Bladder Carcinogens occurring during the Production of "Town" Gas by Coal Carbonization. Paper read to the International Conference on Industrial Medicine, Vienna, July 1966.

Baum, (1874) Third congress of the German Surgical Association, cited by Henry (1946).

Bidstrup, P. L. (1951) Carcinoma of Lung in Chromate Workers. *Br. J. industr. Med.*, **8**, 302.

Bidstrup, P. L. & Case, P. A. M. (1956) Carcinoma of the Lung in Workmen in the Bichromates-producing Industry in Great Britain. *Br. J. industr. Med.*, **13**, 260.

Boyd, J. T., Doll, R., Faulds, J. S. & Leiper, J. (1970) Cancer of the Lung in Iron Ore (Haematite) Miners. *Br. J. industr. Med.*, **27**, 97.

Boyland, E. & Hurning, E. S. (1949) Induction of Tumours with Nitrogen Mustards. *Br. J. Cancer*, **3**, 118.

Bruusgaard, A. (1959) Opptreden av visse kreftformer blant gassverkarbeidere. *Tidssker. norske daegeforening*, p. 755.

Case, R. A. M. & Hosker, M. E. (1954) Tumour of the Urinary Bladder as an Occupational Disease in the Rubber Industry in England and Wales. *Br. J. prev. soc. Med.*, **8**, 39.

Case, R. A.M., Hosker, M. E., McDonald, D. B. & Pearson, J. T. (1954) Tumour of the Urinary Bladder in Workmen Engaged in the Manufacture and use of Certain Dyestuff Intermediates in the British Chemical Industry. *Br. J. industr. Med.*, **11**, 75.

Case, R. A. M. & Lea, A. J. (1955) Mustard Gas Poisoning Chronic Bronchitis and Lung Cancer. *Br. J. prev. soc. Med.*, **9**, 62.

Christian, H. A. (1962) Cancer of the Lung in Employees of a Public Power Plant. A Fifteen Year Study (1946–60). *J. occup. Med.*, **4**, 133.

Cook, J. W., Hewett, C. L. & Hieger, I. (1933) The Isolation of a Cancer-producing Hydrocarbon from Coal Tar. *J. chem. Soc.*, **1**, 395.

De Villiers, A. J. & Windish, J. P. (1964) Lung Cancer in a Fluorspar Mining Community. 1. Radiation, Dust and Mortality Experience. *Br. J. industr. Med.*, **21**, 94.

Doll, R. (1952) The Causes of Death among gas-workers with Special Reference to Cancer of the Lung. *Br. J. industr. Med.*, **9**, 180.

Doll, R. (1955) Mortality from Lung Cancer in Asbestos Workers. *Br. J. industr. Med.*, **12**, 81.

Doll, R., Fisher, R. E. W., Gammon, E. J., Gunn, W., Hughes, G. O., Tyrer, F. H. & Wilson, W. (1965) Mortality of Gasworkers with Special Reference to Cancers of the Lung and Bladder, Chronic Bronchitis, and Pneumoconiosis. *Br. J. industr. Med.*, **22**, 1.

Doll, R., Vessey, M. P., Beasley, R. W. R., Buckley, A. P., Fear, E. O., Fisher, P. E. W., Gammon, E. J., Gunn, W., Hughes, G. O., Lee, K. & Norman-Smith, B. (1972) The Mortality of Gas-workers—Final Report of a Prospective Study. *Br. J. industr. Med.*, **29**, 394.

Duggan, M. J., Soilleux, P. J., Strong, J. C. & Howell, D. M. (1970) The Exposure of United Kingdom Miners to Radon. *Br. J. industr. Med.*, **27**, 106.

Earle, J. (1790) *Chirurgical Works of Percival Pott. A New Edition*. London.

Faulds & Stewart (1956).

Figueroa, W. G., Raszkowski, R. & Weiss, W. (1973) Lung Cancer in Chloromethyl Methyl Ether Workers. *New Engl. J. Med.*, **288**, 1096.

GILSON, J. C. (1966) Health Hazards of Asbestos: Research Studies on its Biological Effects. *Trans. Soc. occup. Med.*, **16**, 62.

GLOYNE, S. R. (1951) Pneumoconiosis: Histological Survey of Necropsy Material in 1205 Cases. *Lancet*, i, 810.

HÄRTING, F. H. & HESSE, W. (1879) Der Lungenkrebs die Bergkrankheit in den Schneeburger. Gruben. *Vjschr. gesichtl. Med.*, *N.S.*, **31**, 102, 313.

HENRY, S. A., KENNAWAY, E. L. & KENNAWAY, N. M. (1931) The Incidence of Cancer of the Bladder and Prostate in Certain Occupations. *J. Hyg. Camb.*, **31**, 125.

HESTON, W. E. (1953) Occurrence of Tumors in Mice Injected Subcutaneously with Sulfur Mustard and Nitrogen. *J. natn. Cancer Inst.*, **14**, 131.

HILL, A. B. (1939) Report to Mond Nickel Company. cited by Morgan (1958).

HILL, A. B. & FANNING, E. L. (1948) Studies in the Incidence of Cancer in a Factory Handling Inorganic Compounds of Arsenic: I. Mortality Experience in the Factory. *Br. J. industr. Med.*, **5**, 1.

HIRSH, A. (1886) *Handbook of Geographical and Historical Pathology* (translated from the second German edition). London: The New Sydenham Society.

HOFFMAN, F. L. (1915) *The Mortality from Cancer Throughout the World.* New Jersey: Prudential Press.

HUEPER, W. C. (1942) *Occupational Tumours.* Baltimore: Thomas.

KENNAWAY, N. M. & KENNAWAY, E. L. (1938) A Study of the Incidence of Cancer of the Lung and Larynx. *J. Hyg. Camb.*, **36**, 236.

KENNAWAY, E. L. & KENNAWAY, N. M. (1944) The Relation between the Incidence and Incubation Period of Cancer in Man. *Yale J. biol. Med.*, **17**, 139.

KURODA, S. & KAWAHATA, K. (1936) Über die gewerbliche Enstehung des Lungenkrebes bei Generatorgas arbeitern. *Z. Krebsforsch.*, **45**, 36.

LASKIN, S., KUSCHNER, M., DREW, R. T., CAPPIELLO, V. P. & NELSON, N. (1971) Tumours of the Respiratory Tract Induced by Inhalation of bis (chloromethyl) ether. *Archs environ. Hlth*, **23**, 135.

LAWTHER, P. J., COMMINS, B. T. & WALLER, R. E. (1965) A Study of the Concentration of Polycyclic Aromatic Hydrocarbons in Gas Works Retort Houses. *Br. J. industr. Med.*, **22**, 13.

LLOYD, J. W. (1971) Long-term Mortality Study of Steelworkers: V. Respiratory Cancer in Coke Plant Operators. *J. occup. Med.*, **13**, 53.

LYNCH, K. M. & SMITH, W. A. (1935) Pulmonary Asbestosis: Carcinoma of Lung in Asbesto-silicosis. *Am. J. Cancer*, **24**, 56.

MALTONI, G. & LEPEMINE, G. (1974) Carcinogenicity Bioassays of Vinyl-Chloride. I. Research Plan and Early Results. *Environm. Res.*, **7**, 387.

MELICK, W. F., ESCUE, H. M., NARYKA, J. J., MEZERA, R. A. & WHEELER, E. P. (1955) First Reported Cases of Human Bladder Tumour due to New Carcinogen-xenylamine. *J. Urol.*, **74**, 760.

MEREWETHER, E. R. A. (1949) *Annual Report of the Chief Inspector of Factories for the Year 1947.* London: H.M.S.O.

MORGAN, J. G. (1958) Some Observations on the Incidence of Respiratory Cancer in Nickel Workers. *Br. J. industr. Med.*, **15**, 224.

NEUBAUER, O. (1947) Arsenical Cancer: a Review. *Br. J. Cancer.*, **1**, 192.

NEWCOMBE, H. B. (1974) Record Linkage for Studies of Environmental Carcinogenesis. *Proc. Tenth Can. Cancer Conf.*, 1973. Toronto: University of Toronto Press. p. 49.

OSBURN, H. S. (1969) Lung Cancer in a Mining District in Rhodesia. *S. Afr. med. J.*, **43**, 1307.

PARIS, A. (1829) *Pharmacologia* 6th edn., **2**, p. 96.

PERRY, K., BOWLER, R. G., BUCKELL, H. M., DRUETT, H. A. & SCHILLING, R. S. F. (1948) Studies in the Incidence of Cancer in a Factory Handling Inorganic Compounds of Arsenic. II. Clinical and Environmental Investigations. *Br. J. industr. Med.*, **5**, 7.

POTT, P. (1775) *Chirurgical Observations relative to the Cataract, Polypus of the Nose, the Cancer of the Scrotum, the Different Kinds of Ruptures and the Mortification of the Toes and Feet.* London.

RASZKOWSKI & WEISS (1973).

ROCKSTROH, H. (1959) Zur Ätiologie des Bronchialkrebses in arsenverabeitenden Nickelhütten. *Arch. Geschwultzforsch.*, **14**, 151.

ROSDAHL, H., LARSEN, S. O. & CLEMMESEN, O. (1974) Hodgkin's Disease in Patients with Previous Infectious Mononucleosis: 30 years' Experience. *Br. med. J.*, ii, 253.

ROTH, F. (1956) Über die chronische Arsenvergiftung der Moselwinzer unter besonderer Berücksichtigung des Arsenkrebses. *Ztschr. Krebsforsch.*, **61**, 287.

SYME, J. (1835) Clinical Report for the Winter Session 1834–35: Chimney Sweepers' Cancer—Excision of the Scrotum—Recovery. *Edinb. med. surg. J.*, **44**, 13.

TUCKER, A. (1975) Figures show Leukaemia Link with Plutonium Workers. *Guardian*, 13 January 1975.

UHLIG, M. (1921) Über den Schneeberger Lungenkrebs. *Vicrhows Arch.*, **230**, 76.

VOLKMANN, R. (1874) Über Theer- und Russkrebs. *Verh. Deut. Gesselsch. Chir.*, **1**, 3.

VAN DUUREN, B. L., GOLDSCHMIDT, B. M. & KATZ, B. S. *et al.* (1968) A New Type of Alkylating Carcinogen. *Archs environm. Hlth*, **16**, 472.

WADA, S., MYANISHI, M., NISHIMOTO, K., KAMBE, S. & MILLER, R. (1968) Mustard Gas as a Cause of Respiratory Neoplasia in Man. *Lancet*, i, 1161.

WADA, S., NISHIMOTO, Y., MIYANISHI, M., KATSUTA, S., NISHIKI, M., YAMADA, A., TOKVOKA, S., UMISA, H. & NAGAI, M. (1962) Review of Okunojima poison Gas Factory regarding Occupational Environment. *Hiroshima J. med. Sci.*, **11**, 75.

WAGNER, J. C., SLEGGS, C. A. & MARCHAND, P. (1960) Diffuse Pleural Mesothelioma and Asbestos Exposure in the North Western Cape Province. *Br. J. industr. Med.*, **17**, 260.

WALPOLE, A. L., WILLIAMS, M. H. C. & ROBERTS, D. C. (1954) Tumour of Urinary Bladder in Dogs after Injection of 4-aminodiphenyl. *Br. J. industr. Med.*, **11**, 105.

YAMAGIWA, K. & ISHIKAWA, K. (1918) Experimental Study of the Pathogenesis of Carcinoma. *J. cancer Res.*, **3**, 1.